# Central diastolic blood pressure, plasma aldosterone and uric acid are associated with microalbuminuria in essential hypertension: a case-control study

**DOI:** 10.1186/s12872-023-03515-1

**Published:** 2023-10-17

**Authors:** Jinlong Li, Ning Yang, Hongda Chou, Leilei Shi, Maoti Wei, Yuming Li

**Affiliations:** 1 Department of Hypertension, Clinical School of Cardiovascular Disease, Tianjin Medical University, Tianjin, 300457 China; 2 Department of Hypertension, TEDA International Cardiovascular Hospital, Tianjin, 300457 China; 3 Center for Clinical Epidemiology, TEDA International Cardiovascular Hospital, Tianjin, 300457 China

**Keywords:** Central blood pressure, Plasma aldosterone, Uric acid, Essential hypertension, Microalbuminuria

## Abstract

**Objective:**

To study the development of microalbuminuria (MAU) in essential hypertension (EHT), we investigated the association of MAU with central blood pressure (CBP), direct renin concentration (DRC), plasma aldosterone (PA), and uric acid (UA).

**Method:**

We determined 24 h-urinary albumin excretion (24 h-UAE) in patients with EHT who were hospitalized at TEDA International Cardiovascular Hospital from June 2020 to May 2022. We defined MAU as 24 h-UAE in the range of 30 mg/24 h to 300 mg/24 h. Univariate and multivariate analyses were conducted to determine the associations of MAU with CBP, DRC, PA, and UA in EHT, considering demographic and clinical information. We also plotted receiver operating characteristic curves (ROCs) for predicting MAU using these results.

**Results:**

More than a quarter of patients (26.5%, 107/404, 95% CI: 22.2–31.1%) were diagnosed with MAU in EHT. A higher body mass index (BMI), longer duration of hypertension, and higher severity were associated with MAU. Also, nearly 10% more creatinine levels were recorded in the MAU group than in the control group (69.5 ± 18.7 µmol/L vs. 64.8 ± 12.5 µmol/L, P = 0.004). The increase was also observed for PA (15.5, 9.7–20.6 ng/dL vs. 12.3, 9.0–17.3 ng/dL, P = 0.024) and UA (419.8 ± 105.6 µmol/L vs. 375.1 ± 89.5 µmol/L, P < 0.001) in the MAU group compared to that in the control group. Several variables were associated with MAU, including central diastolic blood pressure (CDBP) (OR = 1.017, 95% CI: 1.002–1.032, P = 0.027), PA (OR = 1.043, 95% CI: 1.009–1.078, P = 0.012) and UA (OR = 1.005, 95% CI: 1.002–1.008, P < 0.001). For MAU prediction, the area under the curve (AUC) was 0.709 (95% CI: 0.662–0.753; P < 0.001) when CDBP, PA, and UA were used in combination, and the optimal probability of the cut-off value was 0.337.

**Conclusion:**

We found that CDBP, PA, and UA, used for MAU prediction, might be associated with its development during EHT.

**Supplementary Information:**

The online version contains supplementary material available at 10.1186/s12872-023-03515-1.

## Introduction

Hypertension is a global public health concern with a high prevalence and a low rate of diagnosis and control [1]. Complications caused by chronic hypertension are major health risks for the population. It can cause problems such as hypertensive renal injury, which is hidden and can lead to renal failure in the late stages. Hence, early detection and intervention of hypertensive renal injury are important [2].

Several studies have shown that CBP is a good indicator of pressure without resistance of vessels compared to peripheral blood pressure(BP) [3], which can act as a reliable predictor of target organ damage, including central systolic blood pressure (CSBP), CDBP, and central pulse pressure (CPP). CPP is strongly associated with adverse cardiovascular events [4], contrast-induced nephropathy [5], and diabetic kidney damage [6]. CSBP and CDBP are associated with alterations in the carotid artery intima-media thickness, MAU, and left ventricular hypertrophy [7]. However, which indicator is more closely associated with MAU in EHT is not known.

The prevalence of hyperuricemia is increasing every year [8]. In the clinical phase of the disease, urate deposition can cause UA nephropathy, and a decrease in renal function leads to a decrease in the rate of UA excretion. Although many studies have been conducted, the relationship between UA and renal injury in the subclinical phase remains debatable. Some studies have found a positive correlation between increased UA and increased MAU, while others have found a negative relationship [9, 10].

More severe renal damage occurs in primary aldosteronism, and some studies have shown that the aldosterone/renin ratio (ARR) can also predict renal damage in EHT [11]. This finding is also suggested by the stronger renoprotective effect of renin-angiotensin-aldosterone system (RAAS) inhibitors than that exhibited by other classes of antihypertensive drugs.

Considering that CBP, DRC, PA, and UA are associated with renal injury, elucidating the relationship between these factors and MAU in EHT might provide new information. MAU occurs before the decrease in the estimated glomerular filtration rate (eGFR) [12]. In this study, we investigated the relationship between CBP, DRC, PA, UA and 24 h-MAU in hospitalized hypertensive patients.

## Methods

### Study population

Hypertensive patients hospitalized at the Hypertension Center of TEDA International Cardiovascular Hospital from June 2020 to May 2022 were included in this study. Concerning the inclusion criteria, the patients needed to meet the diagnostic criteria of hypertension and an ad libitum sodium diet. Additionally, patients previously under antihypertensive medication (beta-blockers, angiotensin-converting enzyme inhibitors, angiotensin II receptor blockers, and diuretics) needed to be switched to diltiazem terazosin for at least four weeks. The exclusion criteria were as follows: (1) Patients with definite secondary hypertension factors; (2) Patients with severe cardio-cerebrovascular complications (acute heart failure, acute myocardial infarction, acute cerebral infarction, acute cerebral hemorrhage, etc.); (3) Missing data on CBP, 24 h-UAE, DRC, and PA; (4) Patients with 24 h-UAE ≥ 300 mg/24 h; (5) Patients with previous kidney diseases (renal surgery, congenital renal structural malformations, glomerulonephritis, nephrotic syndrome, polycystic kidney disease, etc.); (6) Patients using glucocorticoids, spironolactone, or contraceptives were excluded since they might affect the DRC and PA; (7) Patients with acute infection within two weeks.

This study was conducted in accordance with the ethical standards of the declaration of Helsinki. The study was approved by the Ethics Committee of TEDA International Cardiovascular Hospital Research Project. The need for informed consent was waived by the Ethics Committee of TEDA International Cardiovascular Hospital Research Project as this was a retrospective study.

### General information and collection of laboratory data

The baseline characteristics and medication histories of the patients were collected by hypertension specialists. The laboratory data on white blood cells (WBCs), red blood cells (RBCs), hemoglobin (HGB), fasting glucose, sodium and 24 h-urinary sodium, total cholesterol (TC), triglyceride (TG), high-density lipoprotein cholesterol (HDL), low-density lipoprotein cholesterol (LDL), creatinine, UA, DRC, PA, and 24 h-UAE were collected. The above blood tests and urine-related tests were conducted by the laboratory examination department of TEDA International Cardiovascular Hospital.

### DRC, PA measurement and ARR calculation

Blood collection conditions: (1) collection time: 08:00 a.m., after maintaining a non-reclining position (can be sitting, standing, or walking) for 2 h; the individual was maintained in a sitting position for 10 min, and then the blood sample was collected. (2) Blood was collected carefully to avoid hemolysis. (3) The blood sample to be measured for DRC was kept at room temperature during delivery. The PA and DRC were measured via a fully automated chemiluminescence immunoassay [13]. ARR is the ratio of PA to DRC.

### CBP and BP measurement

The SphygmoCor-XCEL (Version 1.2.0.7; AtCor Medical, Australia) was used for measuring CBP. It is the internationally recognized gold standard for the non-invasive evaluation of CBP. The patient was prepared for the examination depending on the requirements. The patient was asked to lay flat on the examination bed with the right upper limb externally rotated and abducted at 45° to the torso. A suitable cuff was placed in the center of the brachial artery of the exposed upper arm. The arm was positioned in a way that the cuff was at the same level as the heart. CSBP, CDBP, and CPP were then obtained using the software. We measured systolic blood pressure (SBP), diastolic blood pressure (DBP), and pulse pressure (PP) in the right brachial artery with calibrated electronic sphygmomanometer following a standardized protocol and hypertension guidelines.

### Calculation of eGFR and BMI

The modified and simplified MDRD equation was used to calculate eGFR [14]. The modified MDRD equation can be represented as follows: eGFR = 175× Scr^− 1.234^× Age^− 0.179^[×0.79 (female)]. BMI was calculated as body weight in kilograms divided by the square of body height in meters.

### Definition

Smoking was defined as smoking at least 1 cigarette/d for at least six months. Alcohol consumption was defined as alcohol consumption of at least 1 drink/week for at least six months. Hypertension was defined as the self-reported history of hypertension and/or administration of antihypertensive medication and/or elevated BP on the day of investigation; the mean value of three measurements of SBP ≥ 140 and/or the mean value of DBP ≥ 90 mm Hg (1 mm Hg = 0.133 kPa). MAU was defined as 30 mg/24 h ≤ 24 h-UAE < 300 mg/24 h.

All patients were divided into two groups according to 24 h-UAE, the control group (24 h-UAE < 30 mg/24 h), and the MAU group (in the range of 30 mg/24 h to 300 mg/24 h).

### Statistical analysis

Continuous variables with normal distribution were expressed as the mean ± SD and the median with P25–P75 was used to express the data that were not normally distributed. Student’s t-tests or non-parametric tests were conducted to compare data between groups. Paired t-test was used to compare the differences in CBP and BP. Categorical data were represented by numbers (%), and the χ^2^-test was used for comparing categorical data between groups.

After the logarithmic calculation of 24 h-UAE was performed, the correlation of CBP, DRC, PA, and UA with 24 h-UAE and MAU was evaluated. In multivariate logistic regression, gender, age, and variables that were significant in the univariate analysis (BMI, duration of hypertension, grade 3 hypertension, WBC, creatinine, DRC, PA, UA, CSBP and CDBP) were included for adjusting. The variable screening method was backward: conditional, the probability of a variable entering the equation was 0.05, excluding 0.10, and the other parameters were system default. The ROC curves were used to determine the value of CDBP, PA, and UA in predicting MAU in EHT, and the differences between the AUC of each predictor were evaluated for distinguishing between the MAU and control group. All statistical analyses were performed using IBM SPSS25.0 and MedCalc19.0.

## Results

Among 404 EHT patients (38.1 ± 7.8 years old and 78% male), more than a quarter of patients (26.5%, 107/404, 95% CI: 22.2–31.1%) were diagnosed with MAU. Disease histories showed that the EHT were with low prevalence of diabetes, coronary heart disease and cerebrovascular disease (all < 3%). The level of UA was 386.8 ± 95.3 µmol/L and kidney function (eGFR) was 131.4 ± 28.7 mL·min^–1^·1.73 m^–2^. According to eGFR (< 90 mL·min^–1^·1.73 m^–2^), 4.4% of patients could be considered to have reduced kidney function. The CSBP was around 10 mmHg lower than SBP (136.9 ± 17.8 mmHg vs. 146.8 ± 17.1 mmHg, P < 0.001), and CPP was approximately 14 mmHg lower than PP (40.6 ± 8.7 mmHg vs. 54.8 ± 12.3 mmHg, P < 0.001). However, CDBP was higher than DBP (96.3 ± 13.5 mmHg vs. 92.1 ± 13.1 mmHg, P < 0.001).

The MAU group had a higher BMI (28.2 ± 4.2 kg/m^2^ vs. 26.6 ± 3.6 kg/m^2^, P < 0.001), longer duration of hypertension (P = 0.019), and a higher proportion of grade 3 hypertension (78.5% vs. 51.5%, P < 0.001) than the control group. Nearly 10% more creatinine was recorded in the MAU group than that in the control group (69.5 ± 18.7 µmol/L vs. 64.8 ± 12.5 µmol/L, P = 0.004). The MAU group also had higher values of PA (15.5, 9.7–20.6 ng/dL vs. 12.3,9.0–17.3 ng/dL, P = 0.024) and UA (419.8 ± 105.6 µmol/L vs. 375.1 ± 89.5 µmol/L, P < 0.001) than the control group. Also, WBC (6.7 ± 1.8 × 10^9^/L vs. 6.2 ± 1.6 × 10^9^/L, P = 0.025), CSBP (144.5 ± 20.3 mmHg vs. 134.6 ± 16.2 mmHg, P < 0.001), CDBP (102.6 ± 15.8 mmHg vs. 94.1 ± 12.1 mmHg, P < 0.001), and 24 h-UAE (53, 35–82 mg/24 h vs. 12,8.4–17 mg/24 h, P = 0.024) were significantly higher in the MAU group than that in the control group (Table 1).

**Table 1:** Baseline characteristics of patients with and without MAU

Variables	MAU group (n = 107)	Control group (n = 297)	P value
Age, years	38.1 ± 7.6	38.1 ± 7.8	0.994
Male, n (%)	88(82.2)	227(76.4)	0.214
BMI, kg/m^2^	28.2 ± 4.2	26.6 ± 3.6	< 0.001
Smoking, n (%)	37(34.5)	110(37.0)	0.651
Alcohol intake, n (%)	17(15.9)	38(12.8)	0.424
Duration of hypertension, months	24(4,81)	12(2,48)	0.019
Grade 3 hypertension, n (%)	84(78.5)	153(51.5)	< 0.001
Diabetes mellitus, n (%)	1(0.9)	7(2.3)	0.366
Cerebrovascular disease, n (%)	1(0.9)	4(1.3)	0.741
CHD, n (%)	1(0.9)	2(0.7)	0.788
WBC,10^9^/L	6.7 ± 1.8	6.2 ± 1.6	0.025
RBC,10^9^/L	4.9 ± 0.2	4.9 ± 0.4	0.321
HGB, g/L	150.3 ± 15.8	148.7 ± 15.3	0.489
Sodium, mmol/L	141.1 ± 1.9	140.9 ± 1.8	0.181
24 h-urinary sodium, mmol/L	153.3 ± 65.3	147.6 ± 58.8	0.406
Fasting glucose, mmol/L	5.0 ± 0.8	4.9 ± 1.1	0.348
TC, mmol/L	4.7 ± 0.9	4.6 ± 0.8	0.122
TG, mmol/L	2.1 ± 1.5	1.9 ± 1.3	0.135
LDL-C, mmol/L	3.1 ± 0.8	3.0 ± 0.7	0.214
HDL-C, mmol/L	1.1 ± 0.2	1.1 ± 0.2	0.664
Creatinine, µmol/L	69.5 ± 18.7	64.8 ± 12.5	0.004
DRC, µIU/mL	17.5(9.5,37.7)	20.1(10.9,33.3)	0.905
PA, ng/dL	15.5(9.7,20.6)	12.3(9.0,17.3)	0.024
ARR	0.8(0.4,1.4)	0.6(0.4,1.2)	0.280
UA,µmol/L	419.8 ± 105.6	375.1 ± 89.5	< 0.001
Resting heart rate, bpm	77.0 ± 9.7	78.0 ± 11.8	0.365
SBP, mmHg	148.9 ± 19.6	146.4 ± 16.2	0.203
DBP, mmHg	93.2 ± 14.8	91.8 ± 12.5	0.288
PP, mmHg	55.4 ± 13.5	54.5 ± 11.9	0.520
CSBP, mmHg	144.5 ± 20.3	134.6 ± 16.2	< 0.001
CDBP, mmHg	102.6 ± 15.8	94.1 ± 12.1	< 0.001
CPP, mmHg	41.9 ± 8.1	40.3 ± 8.9	0.083
AP, mmHg	10.5 ± 5.5	9.95 ± 9.1	0.547
AI	24.5 ± 10.6	22.5 ± 12.9	0.186
24 h-UAE, mg/24 h	53(35,82)	12(8.4,17)	< 0.001
eGFR, ml·min^− 1^·1.73 m^− 2^	127.3 ± 32.6	132.8 ± 27.1	0.092

Abbreviations: MAU, microalbuminuria; BMI, body mass index; CHD, coronary heart disease; WBC, white blood cell; RBC, red blood cell; HGB, hemoglobin; TC, total cholesterol; TG, triglycerides; LDL-C, low density lipoprotein-cholesterol; HDL-C, high density lipoprotein-cholesterol; DRC, direct renin concentration; PA, plasma aldosterone; ARR, plasma aldosterone/renin ratio; UA, uric acid; SBP, systolic blood pressure; DBP, diastolic blood pressure; PP, pulse pressure; CSBP, central systolic blood pressure; CDBP, central diastolic blood pressure; CPP, central pulse pressure; AP, augmentation pressure; AI, augmentation index; 24h-UAE, 24 h-urinary albumin excretion; eGFR, estimated glomerular filtration rate

**Fig. 1:**
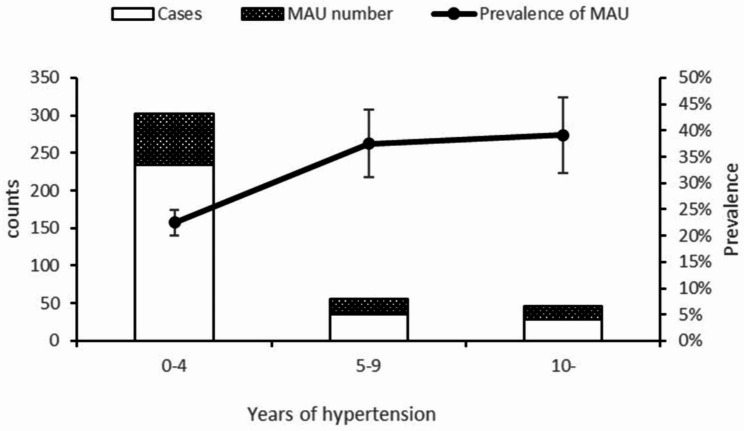
Temporal link between the incidence of MAU and hypertension

We assessed the temporal link between the incidence of MAU and hypertension. The incidence of MAU increased as the duration of hypertension increased(χ^2^_trend_ = 8.761, P = 0.003)(Fig. 1). The prevalence of MAU in hypertension was 22.5% (68/302, 95% CI: 17.9–21.6%) for 0–4 years, 37.5% (21/56, 95% CI: 24.9–51.4%) for 5–9 years, and 39.1% (18/46, 95% CI: 25.1–54.6%) for more than 10 years. Compared to MAU in patients with hypertension for 0–4 years, the prevalence of MAU was significantly higher in patients with hypertension for 5–9 years (χ^2^ = 5.678, P = 0.017) and more than 10 years (χ^2^ = 922, P = 0.015). The incidence of MAU in patients with hypertension for 5–9 years was not significantly different from that in patients with hypertension over 10 years (χ^2^ = 0.028, P = 0.866).

### The correlation between CBP, DRC, PA, UA and Log 24 h-UAE

A linear regression analysis was performed to estimate the correlation between CBP, DRC, PA, UA, and Log 24 h-UAE. The results indicated that CSBP (β = 0.008, P < 0.001), CDBP (β = 0.010, P = 0.011), CPP (β = 0.009, P < 0.001), DRC (β = 0.002, P < 0.001), PA (β = 0.010, P < 0.001), and UA (β = 0.001, P = 0.001) were significantly related to Log 24 h-UAE (Table S1).

### The relationship between CBP, DRC, PA, UA and MAU

A logistic regression analysis was performed to determine the relationship between CBP, DRC, PA, UA, and MAU. The results of the univariate logistic analysis showed that CSBP (OR = 1.031, 95% CI: 1.018–1.045, P < 0.001), CDBP (OR = 1.046, 95% CI: 1.028–1.064, P < 0.001), DRC (OR = 1.009, 95% CI: 1.002–1.016, P = 0.015), PA (OR = 1.042, 95% CI: 1.013–1.072, P = 0.004), and UA (OR = 1.005, 95% CI: 1.002–1.007, P < 0.001) were significantly associated with MAU (Table S2).

After adjusting confounding factors (age, gender, BMI, duration of hypertension, grade 3 hypertension, WBC, creatinine, DRC, PA, UA, CSBP, and CDBP), CDBP (OR = 1.029, 95% CI: 1.009–1.049, P = 0.004), PA (OR = 1.044, 95% CI: 1.010–1.079, P = 0.010), and UA (OR = 1.005, 95% CI: 1.002–1.008, P = 0.001) were still significantly associated with MAU. Other related factors for MAU were grade 3 hypertension (OR = 2.229, 95% CI: 1.267–3.920, P = 0.005) and duration of hypertension (OR = 1.004, 95% CI: 1.000–1.009, P = 0.034) (Table 2).

**Table 2:** The multivariate logistic regression analysis of MAU

Variates	MAU					
	B	SE	Wald χ2	P	OR	95%CI
Duration of hypertension, months	0.004	0.002	4.484	0.034	1.004	1.000 ~ 1.009
Grade 3 hypertension, n (%)	0.801	0.288	7.739	0.005	2.229	1.267 ~ 3.920
PA, ng/dL	0.043	0.017	6.663	0.010	1.044	1.010 ~ 1.079
UA, µmol/L	0.005	0.001	11.886	0.001	1.005	1.002 ~ 1.008
CDBP,mmHg	0.028	0.010	8.099	0.004	1.029	1.009 ~ 1.049

Adjusting confounding factors including age, gender and BMI, duration of hypertension, grade 3 hypertension, WBC, creatinine, DRC,PA,UA,CSBP and CDBP.

Abbreviations: MAU, microalbuminuria ; PA, plasma aldosterone; UA, uric acid; CDBP, central diastolic blood pressure

### Values of CDBP, PA and UA in predicting MAU

**Fig. 2:**
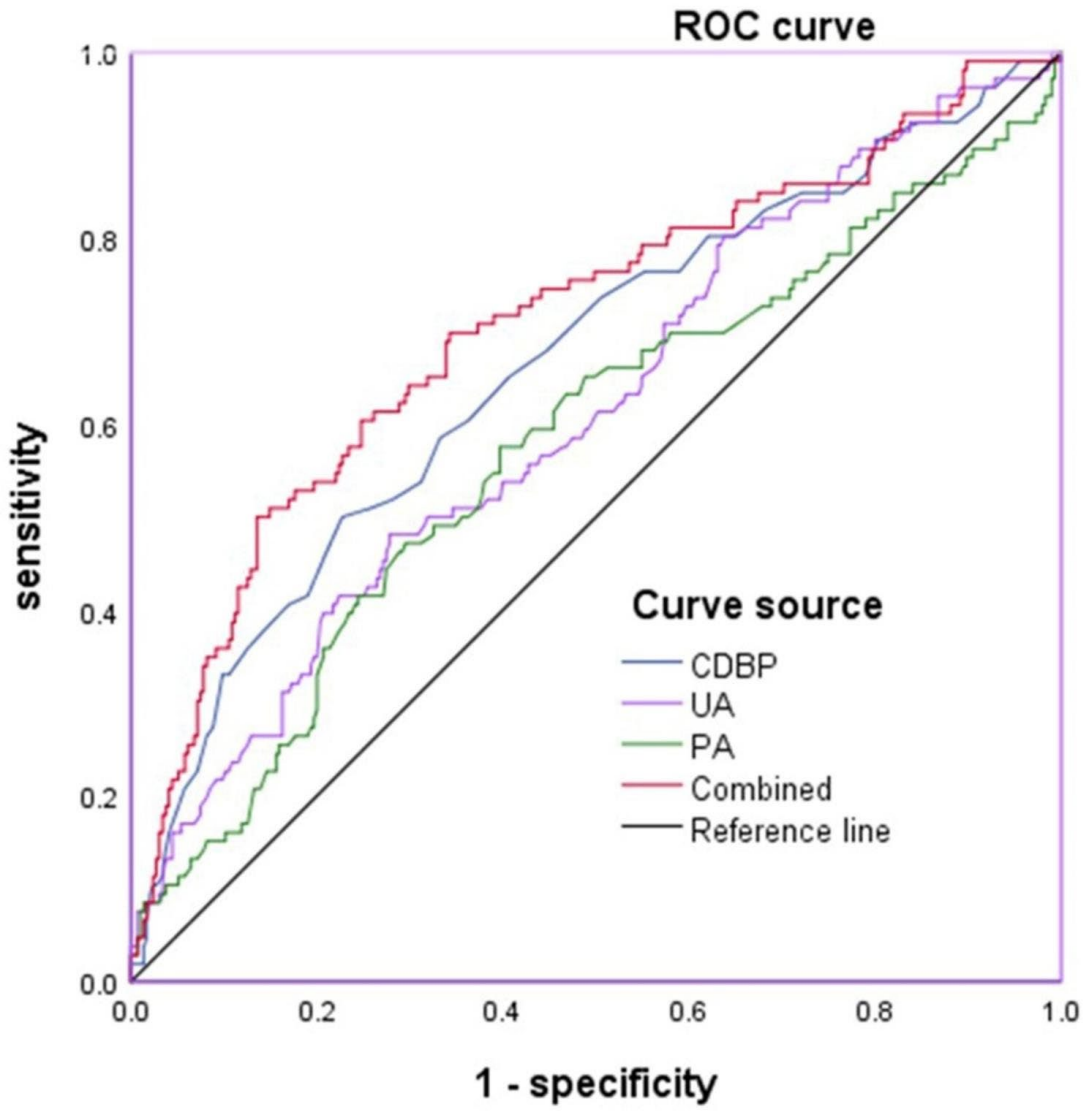
The ROC curves for predicting MAU

The values of CDBP, PA, and UA for predicting MAU were analyzed by the ROC curve (Table 3; Fig. 2). Regarding CDBP, the AUC for predicting MAU was 0.663 (95% CI: 0.614–0.709; P < 0.001). The optimal cut-off value was 101 mmHg with a sensitivity of 49.53% and specificity of 77.44%. For PA, the AUC was 0.574 (95% CI: 0.524–0.622; P = 0.032), and the optimal cut-off value was 14.1 ng/mL with a sensitivity of 57.01% and specificity of 60.27%. For UA, the AUC was 0.613 (95% CI: 0.563–0.661; P < 0.001), and the optimal cut-off value was 421 µmol/L with a sensitivity of 48.11% and specificity of 72.20%. When CDBP, PA, and UA were combined, the AUC was 0.709 (95% CI: 0.662–0.753; P < 0.001). The optimal cut-off value was 0.337, with a diagnostic sensitivity of 50.00% and specificity of 86.44%.

By comparing different ROC curves, we found that all three combined predictions were better than CDBP (P = 0.048), PA (P < 0.001), and UA (P < 0.001) alone.

**Table 3:** ROC curves for predicting MAU

Variates	MAU					
	AUC	P	95%CI	Cut-off value	Sensitivity	Specificity
CDBP	0.663	< 0.001	0.614 ~ 0.709	101mmHg	49.5%	77.4%
UA	0.613	< 0.001	0.563 ~ 0.661	421µmol/L	48.1%	72.2%
PA	0.574	0.032	0.524 ~ 0.622	14.1 ng/dL	57.0%	60.2%
Combined	0.709	< 0.001	0.662 ~ 0.753	0.337	50.0%	86.4%

Abbreviations: MAU, microalbuminuria; CDBP, central diastolic blood pressure; UA, uric acid; PA, plasma aldosterone

## Discussion

Three main findings were highlighted in this study. First, high CDBP and UA are independent risk factors for MAU in EHT. Second, PA, but not DRC and ARR, are independently associated with MAU in EHT. Finally, the combined test of CDBP, PA, and UA can more reliably predict MAU in EHT.

In 2019, the Taiwan Society of Cardiology (TSOC) and the Taiwan Society of Hypertension (THS) developed a consensus for the clinical application of CBP in patients with hypertension, and CBP ≥ 130/90 mmHg was defined as hypertension [15]. A significant inconsistency occurred between BP and CBP in the same individual. CSBP and CPP were lower than SBP and PP, whereas CDBP was higher than DBP. As the age of the patient increased, the values of the two blood pressure parameters became more similar. In this study, the mean age of the participants was 38 years, and the results for CBP and BP were similar to those of other studies. These results also suggested that our data were reliable.

Several studies have shown that CBP might be more relevant than peripheral BP in predicting target organ damage and cardiovascular outcomes [3, 16, 17]. A study on a cohort of 675 patients with hypertension found that CBP was a better predictor of cardiovascular disease than peripheral BP at relatively short follow-ups at ages > 60 years [18]. The same conclusion was reached in studies on children and adolescents [19]. In patients with risk factors for kidney injury, an increase in CSBP was found to increase the risk of developing MAU [7]. In our study, peripheral BP and CBP were correlated with log24 h-UAE. However, the results of the logistic regression analysis of MAU showed that CBP but not peripheral BP was correlated with MAU. It also showed that CBP was a stronger predictor of MAU in EHT.

After correcting for risk factors, we found that CDBP was independently associated with MAU in EHT. Other studies showed similar findings; for example, a cross-sectional study with 1,280 hypertensive patients found that CDBP greater than 90 mmHg was associated with MAU, changes in carotid artery intima-media thickness, and left ventricular hypertrophy [7]. However, some studies have also found that it is CPP that is a predictor of target organ damage. The data from the Strong Heart study suggested that CPP defines the threshold for the risk of cardiovascular risk [20]. A noninvasively-determined CPP above 50 mmHg is strongly related to cardiovascular events. A study with a mean follow-up of 4.8 years (n = 1,426 participants) found that CPP was independently associated with a rapid decline in renal function [21]. In our study, CPP, AP, and AI were not associated with MAU. Further analysis showed that the mean age of the participants and the percentage of combined diabetes mellitus were significantly higher in above studies than in this study. Additionally, the correlation between CBP parameters and different target organ damage (heart, brain, and kidney) was found to be different [16]. This might be the reason for the different results. Our findings suggested that high CDBP might influence MAU in EHT for participants with a relatively low mean age and few comorbidities.

The renin-angiotensin-aldosterone system (RAAS) is the main regulatory system of hemodynamics in humans, and impairments in RAAS strongly affect the development and maintenance of arterial hypertension [22]. In primary aldosteronism, aldosterone is overproduced and renin activity is suppressed [23]. Experimental studies on animals and clinical trials have shown that long-term exposure to an increase in aldosterone levels might result in renal damage independent of blood pressure levels [24, 25]. The eGFR and urinary albumin excretion rate improve with surgery or medication. DRC and PA might be important predictors and therapeutic targets. Studies on patients with non-primary hyperaldosteronism have shown that aldosterone levels are positively correlated with urinary albumin excretion rates [26]. A study proposed the early implementation of aldosterone-targeted therapy in all patients with hypertension and suggested that the guidelines need to be revised [27]. In this study, we found that DRC and PA were associated with log24h-UAE, and after correcting for confounders, PA remained an independent risk factor for MAU, which matched most of the current findings. However, which indicator correlates more strongly with renal injury remains debatable. Mariko et al. found a positive correlation between ARR and urinary albumin excretion and a negative correlation with eGFR in diabetic patients who did not meet the diagnosis of primary hyperaldosteronism [28]. However, they conducted a single-center retrospective study and included only 70 cases. Another retrospective analysis of 275 adolescent and adult patients suggested that in non-primary aldosteronism, ARR predicted metabolic syndrome, obesity, and MAU, and was a better predictor than renin and aldosterone alone [11]. However, more detailed analysis might reveal differences in the 24-h urine sodium results for different subgroups, which might affect the results of the assay. The researchers concluded that prospective studies are needed to determine future screening thresholds.

Most studies could not fully elucidate the relationship between UA and MAU. The Uric Acid Right for Heart Health (URRAH) Project analyzed clinical data of 26,971 individuals and found that UA increased with a decrease in renal function [12]. The prevalence of gout and the frequency of allopurinol use increased significantly with a decrease in eGFR and an increase in albuminuria. Hyperuricemia was found to be independently related to eGFR [12]. A 4.5-year cohort study found that UA is associated with MAU in type 2 diabetes. Thus, early intervention to decrease UA might help in preserving renal function in patients with diabetes [29]. A study conducted a meta-analysis and found that UA-lowering therapy might improve the eGFR and decrease the urinary albumin/creatinine ratio in patients with chronic kidney disease [30]. In our study, UA levels were significantly associated with MAU, which matched the findings of most other studies. However, a study on 409 Chinese adults with a BMI > 24 kg/m^2^ found no significant associations between UA and MAU [31]. The findings of that retrospective study might be related to the small sample size and the fact that the participants did not have any disease. In our study, the causal relationship between UA and MAU in EHT was also not clear. The mediating effects analysis in another study conducted by us showed that high 24 h-UAE was a mediating variable between UA and mildly decreased eGFR [32]. However, the relationship between UA and MAU in EHT needs further investigation.

The URRAH study found a threshold of 4.7 mg/dL for predicting total mortality, 5.6 mg/dL for predicting cardiovascular disease mortality, and 5.7 mg/dL for predicting fatal myocardial infarction [33]. We found that the cut-off value of uric acid for predicting MAU was significantly higher. Further analysis showed that this might be related to the higher proportion of male patients (78%) in our study. The UA levels are generally higher in men than in premenopausal women due to the influence of estrogen.

In hypertensive patients, UA as well as CBP alone has some predictive value in predicting MAU. There have been no studies in which CBP and PA combined with UA predicted MAU in EHT. In the present study, we found that the combination of the three significantly increased the area under the curve and was higher than alone, contributing to the early identification of MAU.

This study had several limitations. (1) After excluding missing data on participants and the data on those who were administered drugs to interfere with RAAS, the number of selected participants, i.e., the sample size, was small. (2) This was a retrospective study, and we could not determine the causal relationship between MAU and related factors. (3) As this was a single-center study, the conclusions might not be applicable to populations from other centers or regions.

In conclusion, we found that CDBP, PA, and UA were associated with MAU in the process of EHT. Their combined application had a better predictive value for MAU in EHT than their single application. Combined testing and intervention targeting might help in the diagnosis and prevention of MAU in EHT.

### Electronic supplementary material

Below is the link to the electronic supplementary material.

Supplementary Material 1

## Data Availability

The datasets used and/or analysed during the current study are available from the corresponding author on reasonable request.

## Abbreviations

EHT: essential hypertension
MAU: microalbuminuria
CBP: central blood pressure
DRC: direct renin concentration
PA: plasma aldosterone
UA: uric acid
ROC: receiver operating characteristic
24 h-UAE: 24 h-urinary albumin excretion
AUC: area under the curve
CI: confidence interval
BMI: body mass index
CDBP: central diastolic blood pressure
BP: blood pressure
CPP: central pulse pressure
CSBP: central systolic blood pressure
RAAS: renin-angiotensin-aldosterone system
eGFR: estimated glomerular filtration rate
WBC: white blood cell
RBC: red blood cell
HGB: hemoglobin
TC: total cholesterol
TG: triglyceride
HDL: high-density lipoprotein cholesterol
LDL: low-density lipoprotein cholesterol
AP: augmentation pressure
AI: augmentation index

## References

[1] Zhou B, Perel P, Mensah GA, Ezzati M (2021). Global epidemiology, health burden and effective interventions for elevated blood pressure and hypertension. Nat Rev Cardiol.

[2] Chen TK, Knicely DH, Grams ME (2019). Chronic kidney disease diagnosis and management: a review. JAMA.

[3] Kollias A, Lagou S, Zeniodi ME, Boubouchairopoulou N, Stergiou GS (2016). Association of Central Versus Brachial blood pressure with target-organ damage: systematic review and Meta-analysis. Hypertension.

[4] Cremer A, Boulestreau R, Gaillard P, Lainé M, Papaioannou G, Gosse P (2018). Twenty-four-hour Central Pulse pressure for Cardiovascular events prediction in a Low-Cardiovascular-Risk Population: results from the Bordeaux Cohort. J Am Heart Assoc.

[5] Huang SS, Huang PH, Leu HB, Wu TC, Lin SJ, Chen JW (2013). Association of central pulse pressure with contrast-induced nephropathy and clinical outcomes in patients undergoing coronary intervention. J Hypertens.

[6] Liu JJ, Liu S, Lee J, Gurung RL, Yiamunaa M, Ang K (2020). Aortic pulse wave velocity, central pulse pressure, augmentation index and chronic kidney disease progression in individuals with type 2 diabetes: a 3- year prospective study. BMC Nephrol.

[7] Bai Y, Wang Q, Cheng D, Hu Y, Chao H, Avolio A (2022). Comparison of risk of Target Organ damage in different phenotypes of arterial stiffness and central aortic blood pressure. Front Cardiovasc Med.

[8] Liu Y, Yan L, Lu J, Wang J, Ma H (2020). A pilot study on the epidemiology of hyperuricemia in chinese adult population based on big data from Electronic Medical Records 2014 to 2018. Minerva Endocrinol.

[9] Ejaz AA, Nakagawa T, Kanbay M, Kuwabara M, Kumar A, Garcia Arroyo FE (2020). Hyperuricemia in kidney disease: a major risk factor for Cardiovascular events, vascular calcification, and renal damage. Semin Nephrol.

[10] Yu X, Gu M, Zhu Y, Zhang L, Kong W, Zou Y (2022). Efficacy of Urate-Lowering therapy in patients with chronic kidney disease: a Network Meta-Analysis of Randomized controlled trials. Clin Ther.

[11] Vecchiola A, Fuentes CA, Barros ER, Martínez-Aguayo A, García H, Allende F (2019). The Aldosterone/Renin ratio predicts Cardiometabolic Disorders in subjects without Classic Primary Aldosteronism. Am J Hypertens.

[12] Russo E, Viazzi F, Pontremoli R, Barbagallo CM, Bombelli M, Casiglia E (2022). Association of uric acid with kidney function and albuminuria: the Uric Acid Right for heArt Health (URRAH) Project. J Nephrol.

[13] Funder JW, Carey RM, Mantero F, Murad MH, Reincke M, Shibata H (2016). The management of primary aldosteronism: case detection, diagnosis, and treatment: an endocrine Society Clinical Practice Guideline. J Clin Endocrinol Metab.

[14] Ma YC, Zuo L, Chen JH, Luo Q, Yu XQ, Li Y (2006). Modified glomerular filtration rate estimating equation for chinese patients with chronic kidney disease. J Am Soc Nephrol.

[15] Cheng HM, Chuang SY, Sung SH, Wu CC, Wang JJ, Hsu PF (2019). 2019 Consensus of the Taiwan Hypertension Society and Taiwan Society of Cardiology on the clinical application of Central Blood pressure in the management of hypertension. Acta Cardiol Sin.

[16] Wang KL, Cheng HM, Chuang SY, Spurgeon HA, Ting CT, Lakatta EG (2009). Central or peripheral systolic or pulse pressure: which best relates to target organs and future mortality. J Hypertens.

[17] Fan F, Qi L, Jia J, Xu X, Liu Y, Yang Y (2016). Noninvasive Central Systolic Blood pressure is more strongly related to kidney function decline than peripheral systolic blood pressure in a Chinese Community-Based Population. Hypertension.

[18] Zuo J, Chang G, Tan I, Butlin M, Chu SL, Avolio A (2020). Central aortic pressure improves prediction of cardiovascular events compared to peripheral blood pressure in short-term follow-up of a hypertensive cohort. Clin Exp Hypertens.

[19] Peluso G, García-Espinosa V, Curcio S, Marota M, Castro J, Chiesa P (2017). High Central Aortic rather than brachial blood pressure is Associated with Carotid Wall Remodeling and increased arterial stiffness in Childhood. High Blood Press Cardiovasc Prev.

[20] Roman MJ, Devereux RB, Kizer JR, Lee ET, Galloway JM, Ali T (2007). Central pressure more strongly relates to vascular disease and outcome than does brachial pressure: the strong heart study. Hypertension.

[21] Xiao W, Wen Y, Ye P, Wang F, Cao R, Bai Y (2020). Noninvasive central pulse pressure is an independent determinant of renal function. J Clin Hypertens (Greenwich).

[22] Viola A, Monticone S, Burrello J, Buffolo F, Lucchiari M, Rabbia F (2015). Renin and aldosterone measurements in the management of arterial hypertension. Horm Metab Res.

[23] Ng E, Gwini SM, Libianto R, Choy KW, Lu ZX, Shen J (2022). Aldosterone, Renin, and Aldosterone-to-renin ratio variability in screening for primary Aldosteronism. J Clin Endocrinol Metab.

[24] Sechi LA, Novello M, Lapenna R, Baroselli S, Nadalini E, Colussi GL (2006). Long-term renal outcomes in patients with primary aldosteronism. JAMA.

[25] Lin X, Ullah M, Wu X, Xu F, Shan SK, Lei LM (2021). Cerebro-Cardiovascular Risk, Target Organ damage, and treatment outcomes in primary Aldosteronism. Front Cardiovasc Med.

[26] Petramala L, Concistrè A, Mezzadri M, Sarlo F, Circosta F, Schina M (2022). Relationship between plasma aldosterone levels and arterial stiffness parameters in hypertensive patients with subclinical vascular damage. Int J Cardiol Cardiovasc Risk Prev.

[27] Pitt B, Vaidya A (2023). Early implementation of Aldosterone-Targeted therapy in patients with hypertension. Circulation.

[28] Higa M, Ichijo T, Hirose T (2022). Aldosterone-to-renin ratio is Associated with Diabetic Nephropathy in type 2 Diabetic Patients: a Single-Center Retrospective Study. Med Sci Monit.

[29] Lai YJ, Chen YY, Ku PW, Chen LJ, Yen YF (2021). Association between uric acid level and incidence of albuminuria in patients with type 2 diabetes mellitus: a 4.5-year cohort study. Med (Baltim).

[30] Tsukamoto S, Okami N, Yamada T, Azushima K, Yamaji T, Kinguchi S (2022). Prevention of kidney function decline using uric acid-lowering therapy in chronic kidney disease patients: a systematic review and network meta-analysis. Clin Rheumatol.

[31] Li L, Song Q, Yang X (2019). Lack of Associations between elevated serum uric acid and components of metabolic syndrome such as hypertension, Dyslipidemia, and T2DM in overweight and obese chinese adults. J Diabetes Res.

[32] Chou H, Wei M, Chen H, Xu Y, Shi L, Duan J (2023). The association among uric acid, microalbumin and estimated glomerular filtration rate in hypertensive patients: a case control study. BMC Cardiovasc Disord.

[33] Maloberti A, Giannattasio C, Bombelli M, Desideri G, Cicero A, Muiesan ML (2020). Hyperuricemia and Risk of Cardiovascular Outcomes: the experience of the URRAH (Uric Acid Right for Heart Health) Project. High Blood Press Cardiovasc Prev.

