# Supplementary material for: Central diastolic blood pressure, plasma aldosterone and uric acid are associated with microalbuminuria in essential hypertension: a case-control study
Source: BMC Cardiovasc Disord. 2023 Oct 17;23:511. doi: 10.1186/s12872-023-03515-1 (PMC10583460; doi:10.1186/s12872-023-03515-1)
Supplement: Supplementary file 1 — Supplementary Material 1 [file 12872_2023_3515_MOESM1_ESM.docx]

Table S1The univariate linear regression analysis for Log 24h-UAE

| Variates | Log 24h-UAE | | | | |
| --- | --- | --- | --- | --- | --- |
|  | B | SE | β | t | P |
| BMI, kg/m^2^ | 0.020 | 0.005 | 0.184 | 3.757 | <0.001 |
| Duration of hypertension, months | 0.001 | 0.000 | 0.115 | 2.322 | 0.021 |
| Grade 3 hypertension, n (%) | 0.210 | 0.042 | 0.243 | 5.027 | <0.001 |
| SBP, mmHg | 0.004 | 0.001 | 0.145 | 2.941 | 0.003 |
| DBP, mmHg | 0.004 | 0.002 | 0.117 | 2.360 | 0.019 |
| WBC,10^9^/L | 0.049 | 0.013 | 0.191 | 3.887 | <0.001 |
| Fasting glucose, mmol/L | 0.054 | 0.021 | 0.127 | 2.556 | 0.011 |
| 24h- urinary sodium, mmol/L | 0.001 | 0.000 | 0.101 | 2.026 | 0.043 |
| TC, mmol/L | 0.067 | 0.027 | 0.124 | 2.489 | 0.013 |
| TG, mmol/L | 0.034 | 0.016 | 0.107 | 2.138 | 0.033 |
| LDL-C, mmol/L | 0.059 | 0.029 | 0.102 | 2.038 | 0.042 |
| Creatinine, μmol/L | 0.005 | 0.001 | 0.171 | 3.474 | 0.001 |
| DRC, μIU/mL | 0.002 | 0.001 | 0.217 | 4.464 | <0.001 |
| PA, ng/dL | 0.010 | 0.003 | 0.181 | 3.687 | <0.001 |
| UA, μmol/L | 0.001 | 0.000 | 0.170 | 3.453 | 0.001 |
| CSBP, mmHg | 0.008 | 0.001 | 0.329 | 6.911 | <0.001 |
| CDBP, mmHg | 0.010 | 0.001 | 0.314 | 6.624 | <0.001 |
| CPP, mmHg | 0.009 | 0.002 | 0.176 | 3.581 | <0.001 |

Abbreviations: 24h-UAE，24h-urinary albumin excretion；BMI, body mass index; SBP, systolic blood pressure; DBP, diastolic blood pressure; WBC, white blood cell; TC, total cholesterol; TG, triglycerides; LDL-C, low density lipoprotein-cholesterol; DRC, direct renin concentration; PA, plasma aldosterone; UA, uric acid; CSBP, central systolic blood pressure; CDBP, central diastolic blood pressure; CPP, central pulse pressure.

Table S2 The univariate logistic regression analysis of MAU

| Variates | MAU | | | | | |
| --- | --- | --- | --- | --- | --- | --- |
|  | B | SE | Wald χ2 | P | OR | 95%CI |
| Age, years | 0.000 | 0.015 | 0.000 | 0.993 | 1.000 | 0.972~1.029 |
| Male, n (%) | -0.356 | 0.288 | 1.537 | 0.215 | 0.700 | 0.399~1.230 |
| BMI, kg/m^2^ | 0.097 | 0.029 | 11.086 | 0.001 | 1.101 | 1.041~1.166 |
| Smoking, n (%) | -0.107 | 0.236 | 0.205 | 0.651 | 0.899 | 0.566~1.427 |
| Alcohol intake, n (%) | 0.253 | 0.316 | 0.638 | 0.425 | 1.287 | 0.692~2.394 |
| Duration of hypertension, months | 0.005 | 0.002 | 6.442 | 0.011 | 1.005 | 1.001~1.009 |
| Grade 3 hypertension, n (%) | 1.235 | 0.262 | 22.138 | <0.001 | 3.437 | 2.055~5.749 |
| Diabetes mellitus, n (%) | -0.939 | 1.075 | 0.764 | 0.382 | 0.391 | 0.048~3.214 |
| Cerebrovascular disease, n (%) | -0.370 | 1.124 | 0.108 | 0.742 | 0.691 | 0.076~6.252 |
| CHD, n (%) | 0.330 | 1.230 | 0.072 | 0.788 | 1.392 | 0.125~15.504 |
| WBC,10^9^/L | 0.150 | 0.067 | 4.952 | 0.026 | 1.162 | 1.018~1.325 |
| RBC,10^9^/L | 0.261 | 0.262 | 0.988 | 0.320 | 1.298 | 0.776~2.169 |
| HGB, g/L | 0.005 | 0.007 | 0.482 | 0.488 | 1.005 | 0.991~1.020 |
| Fasting glucose, mmol/L | 0.098 | 0.105 | 0.863 | 0.353 | 1.013 | 0.897~1.355 |
| TC, mmol/L | 0.220 | 0.143 | 2.388 | 0.122 | 1.246 | 0.943~1.648 |
| TG, mmol/L | 0.117 | 0.079 | 2.174 | 0.140 | 1.124 | 0.962~1.313 |
| Sodium, mmol/L | 0.083 | 0.062 | 1.788 | 0.181 | 1.086 | 0.962~1.226 |
| 24h- urinary sodium, mmol/L | 0.002 | 0.002 | 0.693 | 0.405 | 1.002 | 0.998~1.005 |
| LDL-C, mmol/L | 0.191 | 0.154 | 1.545 | 0.214 | 1.211 | 0.896~1.637 |
| HDL-C, mmol/L | -0.212 | 0.486 | 0.190 | 0.663 | 0.809 | 0.312~2.099 |
| Creatinine, μmol/L | 0.022 | 0.008 | 7.646 | 0.006 | 1.022 | 1.006~1.038 |
| DRC, μIU/mL | 0.009 | 0.004 | 5.936 | 0.015 | 1.009 | 1.002~1.016 |
| PA, ng/dL | 0.041 | 0.015 | 8.174 | 0.004 | 1.042 | 1.013~1.072 |
| ARR | 0.000 | 0.080 | 0.000 | 0.996 | 1.000 | 0.854~1.171 |
| UA, μmol/L | 0.005 | 0.001 | 15.017 | <0.001 | 1.005 | 1.002~1.007 |
| Resting heart rate, bpm | -0.009 | 0.010 | 0.823 | 0.364 | 0.991 | 0.971~1.011 |
| SBP, mmHg | 0.008 | 0.007 | 1.621 | 0.203 | 1.008 | 0.996~1.021 |
| DBP, mmHg | 0.009 | 0.009 | 1.131 | 0.288 | 1.009 | 0.992~1.027 |
| PP, mmHg | 0.006 | 0.009 | 0.416 | 0.519 | 1.006 | 0.988~1.024 |
| CSBP, mmHg | 0.031 | 0.007 | 21.632 | <0.001 | 1.031 | 1.018~1.045 |
| CDBP, mmHg | 0.045 | 0.009 | 25.165 | <0.001 | 1.046 | 1.028~1.064 |
| CPP, mmHg | 0.022 | 0.013 | 2.991 | 0.084 | 1.022 | 0.997~1.049 |
| AP, mmHg | 0.008 | 0.013 | 0.357 | 0.550 | 1.008 | 0.982~1.034 |
| AI | 0.012 | 0.009 | 1.749 | 0.186 | 1.012 | 0.994~1.031 |
| eGFR, ml·min^-1^·1.73 m^-2^ | -0.007 | 0.004 | 2.830 | 0.093 | 0.993 | 0.985~1.001 |

Abbreviations: MAU: microalbuminuria; BMI, body mass index; CHD, coronary heart disease; WBC, white blood cell; RBC, red blood cell; HGB, hemoglobin; TC, total cholesterol; TG, triglycerides; LDL-C, low density lipoprotein-cholesterol; HDL-C, high density lipoprotein-cholesterol; DRC, direct renin concentration; PA, plasma aldosterone; ARR, plasma aldosterone/renin ratio; UA, uric acid; SBP, systolic blood pressure; DBP, diastolic blood pressure; PP, pulse pressure; CSBP, central systolic blood pressure; CDBP, central diastolic blood pressure; CPP, central pulse pressure, AP, augmentation pressure; AI, augmentation index; eGFR: estimated glomerular filtration rate.
